# Reconstruction of Cellular Signal Transduction Networks Using Perturbation Assays and Linear Programming

**DOI:** 10.1371/journal.pone.0069220

**Published:** 2013-07-30

**Authors:** Bettina Knapp, Lars Kaderali

**Affiliations:** 1 Institute for Medical Informatics and Biometry, Medical Faculty Carl Gustav Carus, Technische Universität Dresden, Dresden, Germany; 2 ViroQuant Research Group Modeling, BioQuant, Heidelberg University, Heidelberg, Germany; University of Ulm, Germany

## Abstract

Perturbation experiments for example using RNA interference (RNAi) offer an attractive way to elucidate gene function in a high throughput fashion. The placement of hit genes in their functional context and the inference of underlying networks from such data, however, are challenging tasks. One of the problems in network inference is the exponential number of possible network topologies for a given number of genes. Here, we introduce a novel mathematical approach to address this question. We formulate network inference as a linear optimization problem, which can be solved efficiently even for large-scale systems. We use simulated data to evaluate our approach, and show improved performance in particular on larger networks over state-of-the art methods. We achieve increased sensitivity and specificity, as well as a significant reduction in computing time. Furthermore, we show superior performance on noisy data. We then apply our approach to study the intracellular signaling of human primary nave CD4^+^ T-cells, as well as ErbB signaling in trastuzumab resistant breast cancer cells. In both cases, our approach recovers known interactions and points to additional relevant processes. In ErbB signaling, our results predict an important role of negative and positive feedback in controlling the cell cycle progression.

## Introduction

Functional knockdowns for example by RNA interference (RNAi) are a powerful tool to identify genes involved in a specific biological process. The technology has been widely employed in large scale screening approaches, for example to identify genes relevant for cellular growth and viability, for cell proliferation, in bacterial or viral infection, in signaling, in cellular trafficking, influencing the chemosensitivity of tumors, or determining stem cell identity [1]–[13]. While functional knockdowns are very successful to identify genes associated with a particular phenotype, the spatial and temporal placement of hits in their surrounding signaling or regulatory networks poses considerable challenges [14]. In silico network reconstruction using machine learning methods has been used to infer underlying molecular networks from perturbation data with some success. Approaches suggested include Bayesian [15] or dynamic Bayesian networks [16], probabilistic Boolean threshold networks [17], [18], conditional correlation analysis [19], differential equation models [20] and others. For knockdown data with high-dimensional phenotypes acquired for example using microarrays, Nested Effects Models (NEMs) can be used [21]–[25]. NEMs use the nested structure of phenotypic effects after different knockdowns to infer a hierarchy of genes. The underlying assumption is that if gene A is upstream of gene B in a signaling pathway, then the effects seen after a knockdown of A must be a superset of the effects seen after knockdown of B. While NEMs were recently extended to handle time-course measurements [26], [27], they still have severe limitations when applied to large networks, and they cannot handle combinatorial knockdowns. This, however may be crucial to distinguish between complex network topologies, for example with feed-forward loops. In addition, NEMs require high-dimensional “effects” observations after every knockdown, which are not routinely measured in many perturbation screens. Such phenotypic data furthermore offers only very indirect information about the signaling pathway at hand. Direct observations of protein states cannot be used with NEMs. These limitations were the motivation for the development of Deterministic Effects Propagation Networks (DEPNs) [28]. DEPNs assume deterministic signaling in the underlying network, and introduce noise only at the measurement stage. The measurement distribution of active versus inactive proteins is then estimated from the data either using maximum likelihood inference or maximization of the posterior distribution. Given the measurement distribution and knockdown data, alternative network topologies can then be scored. Dynamic DEPNs (D-DEPNs) have recently been proposed as an extension of DEPNs that explicitly take time course data into account [29].

Besides statistical approaches, also combinatorial optimization methods have been suggested to tackle the problem of inferring a signaling network from perturbation data. Ourfali *et al.* proposed an integer programming approach to infer an integrated protein-protein and protein-DNA interaction network [30]. The authors used gene expression measurements after knockout experiments combined with database information to reconstruct regulatory pathways in yeast. A similar approach has been suggested by Lan *et al.*, linking genetic and transcriptomic screening data with data of known molecular interactions [31]. The drawback of both approaches is that they need a network to start with, hence, these methods cannot be used if no such prior information is available. Furthermore, Hashemikhabir *et al.* recently showed that even when assuming that an approximately correct network is given, finding the minimum number of topological changes to make this network consistent with given experimental data is an NP complete problem [32]. In fact, the exponentially increasing number of possible networks for increasing number of genes is the most important limiting factor when inferring network topologies. For a directed graph over 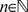 nodes, there are 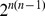 possible network topologies (forbidding cycles of length one). Complete enumeration of the solution space thus quickly becomes infeasible already for 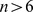 or 7 [17], [21].

In this manuscript, we consider signal transduction as an information flow through a network, that is perturbed by experimental interventions. The idea is to formulate the network inference problem as an integer linear program (ILP), where the 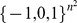 solution vector specifies for each ordered pair of nodes if they are linked by an inhibition, by no interaction, or by an activation. However, ILP is an NP hard problem. We therefore drop the integrality constraint, converting the ILP into a non-integral linear program (LP). Edges then have continuous edge-weights, and the decision whether or not to include an edge into the final network requires a heuristic decision using a threshold-based discretization. This formulation of the network inference problem as a LP allows the use of polynomial time solvers such as the ellipsoid method [33]. We here use the simplex algorithm as LP solver. Albeit this algorithm is not necessarily polynomial, it has proven itself to be very efficient in practice [34].

We have implemented this approach in the R programming language [35]. To demonstrate its application, we evaluate our approach on simulated data, and show that it can robustly deal with noisy and missing data. An evaluation on large scale networks shows an over 10 fold decrease in running time whilst demonstrating superior performance over other current state-of-the-art methods. Using the method on signal transduction downstream of CD3, CD28 and LFA-1 in CD4^+^ T-cells, we demonstrate the applicability to real experimental data. Last but not least, we applied the approach to reconstruct ErbB signaling in breast cancer cells. Our approach could successfully reconstruct known interactions, and furthermore pointed to an important role of feedback loops in regulating the cell cycle progression mediated by the ErbB pathway.

## Methods

Let an (unknown) graph 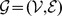 with nodes 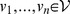 and directed edges 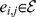, 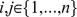 be given. We define the edge 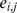 to go from node 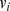 to node 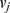. The set of vertices 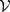 corresponds to proteins or protein complexes, and the set of edges describes activatory or inhibitory interactions between proteins, for example by phosphorylation or dephosphorylation. Each protein 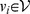 is associated with an activity level 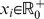, and can either be active (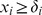) or inactive (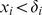). The parameter 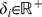 is a positive, node-specific threshold level. Finally, edges 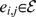 have weights 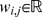 associated with them. Activating edges are characterized by 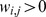, inhibiting edge by 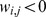. For notational convenience, we write 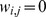 if 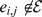. We now make the assumption that
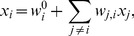
(1)


hence the activity level of a protein 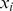 is fully determined by other proteins 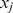 in the network with 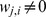. Here, 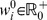 is a bias term that describes the baseline activity of 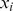 in the absence of any external regulations. We then write
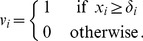
(2)


The graph 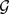 is fixed in our setting, but unknown. We can now experimentally perturb 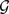 by forcing individual nodes 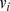 to the *inactive* state 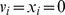, and then observe the influence this has on all other nodes in 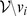.

Our mathematical model of signal transduction is based on the notion of an information flow through 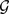. The flow begins at one or several *source nodes* 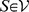 and is then propagated via the edges 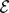 through the network until it reaches one or several *sink nodes* 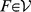. Thus, a protein 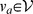 influences another protein 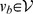 if there exists a directed path from 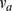 to 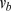. If there is a direct connection 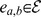 we say that 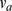 is the parent node of 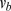 and 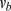 is the child node. According to equation (2), a knockdown of a node 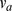 implies that its children may change their activity states. The problem we have to solve is to infer the underlying edge weights 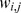 from observations of node activity levels after a set of such perturbation experiments. The only constraint we impose on 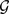 is that 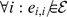. Cycles of length 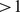 are explicitly permitted.

Now let a set 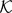 of 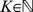 different perturbation experiments be given, where each perturbation experiment 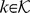 consists of the simultaneous knockdown of one or several nodes in 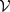. Given experimental data, we define the *observation matrix* 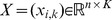, where 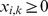 is the observed activity level of 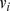 after perturbation experiment 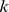. We here use continuous values for 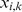, accounting for diverse types of experimental measurements that quantify protein abundance, for example fluorescence measured after antibody staining. We furthermore define the *activation matrix* 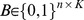 as
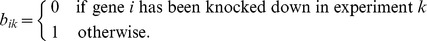



The matrix 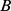 specifies which of the genes in the network were targeted by which knockdown experiment. The respective genes are fixed to the “inactive” state and are no longer subject to regulation by other genes. Since we permit combinatorial knockdowns of multiple genes simultaneously in one experiment, 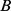 is a 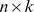 matrix, and not simply an index vector.

### Linear Programming Model

We can now formulate the network inference problem as a linear program. Assuming that biological networks are sparse [36], we minimize the sum of the absolute edge weights 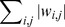 and bias terms 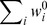. We furthermore introduce slack variables 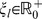, 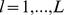 that permit slight violations of constraints of the LP model and can thus account for noise in the experimental data. To minimize the extent to which slack variables are used, we include the sum of the slack variables in the objective function. The variable 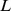 describes the cardinality of the set of inactive genes in the experimental data, 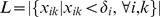, and corresponds to the number of constraints that may be violated in the linear program. The full LP then becomes:
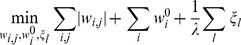
(3)


subject to the constraints
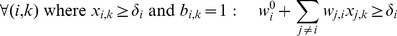
(4)

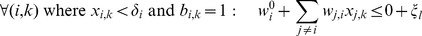
(5)


The constraints (4) and (5) are defined for each pair 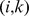 and specify the effect of the knockdown 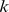 on gene 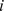. According to equations (1) and (2), for given knockdown 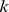, the activity of each gene 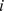 is determined by the activities of its parents 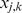, the strength of their influence 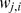, and gene 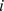
's baseline activity 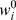. Thus, if gene 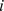 is active after perturbation 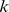, that is, if 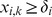, and gene 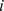 has not been silenced in knockdown 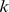 (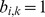), constraint (4) has to hold. Similarly, if the gene is inactive, we require 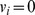, and hence constraint (5) has to hold. We note that we do not need to consider observations 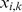 for the 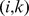 pairs where 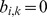, since these correspond to perturbed genes directly targeted by the knockdowns. The respective genes/proteins are thus no longer influenced by incoming regulations. Furthermore, the constraints (4) and (5) relax equation (2) in that equality is no longer required, but instead a margin of 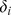 is enforced between activated and non-activated node states.

Missing observations 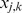 can heuristically be treated in this framework as follows: If in constraints (4) or (5), a variable 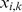 is missing on the left hand side, the constraint is simply left out. If one of the 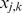 is missing on the right hand side, the corresponding worst-case is assumed, i.e. in case of constraint (4), the missing value is assumed 0, whereas in contraint (5), the missing value is assumed to be 1.

The function of the slack variables is to allow violations of the constraints (5), in case of contradictions between constraints (4) and (5). The parameter 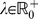 is a non-negative penalty parameter to control the introduction of slack variables 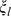 in constraint (5). Intuitively, if 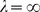, the slack variables can become infinitely large without affecting the objective function (3); conversely, if 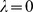, slack variables are not allowed. We use leave-one-out crossvalidation (LOOCV) to choose 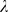 optimally for a given data set. To restrict the introduction of slack variables, we restrict 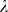 to be at most 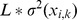, where 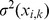 is the variance of the observations 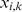 for all 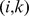. Thus, the higher the variance of the data the higher the slack variables can become. The upper bound is chosen based on the worst case where all 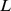 slack variables are unequal to zero.

### Inclusion of Prior Knowledge

In many cases, some knowledge about the biological processes underlying a particular data set will already be given. This can be used to formulate additional constraints, for example requiring certain edge weights 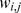 to be above or below a certain threshold if it is known that the respective proteins do or do not interact. Similarly, if it is known which proteins 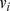 are receptors (source nodes 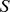) or sink nodes 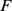, the following additional constraints can be included:
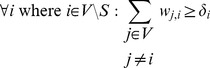
(6)

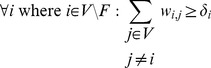
(7)


The constraints force each node that is not a source or sink node to have at least one incoming and one outgoing edge.

### Data Simulation and Network Inference on Simulated Data

To evaluate our model on simulated data, we used network topologies that were taken from the KEGG database [37] as ground truth. We randomly extracted sub-networks from randomly selected KEGG signaling networks, for details see file S1. Only gene-gene interactions in KEGG were considered. We then simulated single knockdowns of every protein 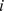 in each of the networks, double knockdowns of 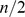 randomly chosen protein pairs, as well as one experiment without any perturbation. Data simulation was done using equations (1) and (2), by setting 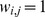 for all edges 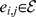. Nodes 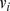 without incoming edges were assumed to have 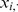 sampled from a Normal distribution with mean 0.95 and standard deviation 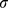, unless 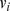 was directly targeted by the knockdown. To then simulate measurement data from the simulated node activities, we employed two Gaussian probability distributions, one for active and one for inactive proteins. Continuous observations of an activated node were simulated from the normal distribution 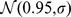, or from 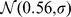 for inactive nodes, in line with the procedure employed by Fröhlich *et al.* [28]. The values of the means in the two Gaussians were chosen to agree with average levels of activatory and inhibitory proteins as we observed in the ErbB data set [28]. The parameter 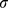 was chosen as described below, 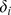 was generated with a normal distribution of 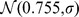. We generated data with three replicates for each type of experiment. For the network inference with the LP model, the replicates have been summarized using the arithmetic average.

To find the best parameter 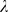 in the range 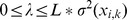 and to compute a range of possible weights for each edge we used LOOCV with a grid search. The basic idea is to leave out parts of the observational data, infer networks on the remaining data with different values of 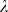, and use the resulting networks to predict activity levels of data that were left out in network inference. This prediction was repeated 100 times, and we calculated every time the MSE between the predicted and observed activity levels. The best parameter 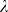 is the one with minimal MSE. For evaluation of resulting networks, we computed the median and the median absolute deviations (MAD) of the edge weights learned in each step. This is necessary, since different weights can be learned for individual interactions in each cross-validation step. We included only robustly learned edges in the final network, requiring that the median of the learned edges from the different crossvalidation runs was larger than the median absolute deviation (MAD) over the runs.

We simulated data based on ten-node networks to evaluate the performance of our approach on noisy and missing data. We furthermore tested how the introduction of prior knowledge improves results. Furthermore, we applied our approach on simulated data of larger networks, to assess performance on bigger problems and to measure how the computational time increases with increasing network size.

### Ten-Node Networks

We extracted ten different networks from KEGG with 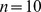 nodes each. The extracted networks have a varying number of edges: five networks have seven interactions, the remaining networks have five, eight, ten, twelve and thirteen interactions, respectively. All edges were assumed positive, i.e. there are no inhibitions in the simulated networks. To assess how our inference approach performs on noisy data, we simulated different noise levels in the generated data by varying 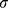 with values of 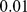, 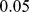, 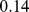 and 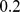. For the evaluation of our model on incomplete data, we randomly selected 10%, 20%, 40% and 50% of the genes and removed all the measurements given for them. Thus, 10% missing values corresponds to one gene without any observations. We repeated data simulation in this way 10 times for each network and each percentage. For the simulation of missing data for the ten-node networks, a noise level of 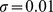 was employed in the two Gaussian distributions describing measurement noise.

Lastly, we tested how the integration of prior knowledge improves network inference. We therefore randomly selected 10%, 25%, 50% and 100% of the true interactions, and included the additional constraints 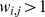 for these edges in the inference. In addition, we separately inferred the networks assuming that the identities of the source and sink nodes are given, but assuming no knowledge about edges, again using 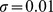.

### Larger Networks

To evaluate the performance of our LP model on larger problems, we extracted five networks from KEGG with 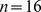, 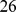, 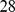, 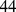 and 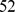 nodes, respectively. As above, we simulated only activatory interactions, with 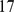, 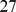, 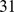, 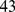 and 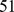 edges in the five networks. We then simulated single knockdowns of every node, 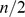 randomly chosen double knockdowns, and one experiment without any knockdown for each of the networks, and reconstructed the underlying networks from the simulated data alone using our LP approach. In contrast to inference on the ten-node networks, we switched from LOOCV to ten-fold crossvalidation for the estimation of 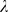. This significantly reduces the number of times the training process is repeated and thus the total run time.

### Evaluation of Inference Results

For both, the ten-node networks and the large-scale problems, we compared our results with those derived with the recently published DEPN approach [28]. For each generated data set, we inferred network topologies using the LP model and the DEPN approach, calculated receiver operating characteristic (ROC) [38], [39] curves of the learned interactions and computed the area under the curve for the ROC-curve (AU-ROC) and the precision-to-recall curve (AU-PR). To assign a weight to each edge for the DEPN approach, we used greedy hillclimbing and bootstrapping (resampling with replacement) with 100 bootstrap samples, as proposed in the DEPN implementation [28]. We furthermore considered only edges appearing with a frequency higher than 0.5 for the evaluation. Since the DEPN approach cannot infer negative interactions, we treated our LP model similarly and ignored the signs of the edge weights.

We note here that there are two different philosophies underlying the networks reconstructed by the DEPN approach and by our method. In the DEPN approach, an edge 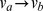 implies that 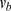 is downstream of 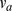 in the network, and will be affected by a knockdown of 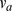. Therefore, DEPNs assume transitivity: If there are edges 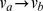 and 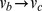, then the DEPNs also infer an edge 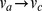, since a knockdown of node 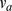 will affect node 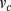 indirectly via 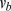. DEPNs thus return equivalence classes of networks, and not a single unique network. Our interpretation is different: We interpret edges as *direct* physical interactions between molecules, and lack of an edge means that there is no direct interaction between the molecules. Edges are then not transitive. This leads to a fundamental difference to the DEPNs: Provided sufficient data are available, a unique minimal network can be inferred from the data. We believe that, in a biological setting, one is usually interested in inferring the actual network of physical interactions, and not a transitively closed network of upstream-downstream relations. We therefore in the following show the performance evaluation based on the actually inferred networks (i.e. we compare the single network inferred by our approach and the transitively closed network returned by the DEPN against the gold standard network). This comparison is biased, since a whole equivalence class is compared against a single network for the DEPN approach. As an alternative, we compared the DEPN results against the transitive closure of the reference network, thus comparing the two equivalence classes; these results are given in figure S1 and table S1.

### Network Inference on Real Data

Simulated data can be used to study the effect of different characteristics of data on network inference performance, however, only an evaluation on real data can provide a realistic picture of the practical applicability of a method. To assess performance of our approach on real world problems, we used two different publicly available data sets: The first data set focuses on the signal transduction downstream of CD3, CD28 and LFA-1 in primary nave CD4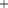 T-cells [15], the second data set considers ErbB signaling in a breast cancer cell line [28]. We compared performance of our approach on both data sets with random guessing and inference using the DEPN approach, and with results of the Bayesian approach employed by Sachs *et al.* in case of the CD4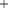 T-cell data [15].

### CD4^+^ T-Cell Signaling after CD3, CD28 and LFA-1 Stimulation

The first data set we used regards an intracellular signaling network in human primary nave CD4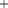 T-cells. This data set was published by Sachs *et al.* in 2005, and comprises nine perturbation experiments (overactivations and inhibitions) with effects quantified using flow cytometry [15]. Given are measurements of the 11 phosphorylated proteins and phospholipids PKC, PKA, Akt, Raf, Mek1/2, Erk1/2, p38, JNK, PIP2, PIP3, PLY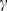 downstream of CD3, CD28 and LFA-1. The perturbation conditions consist of four stimulatory experiments and five inhibitions. Quantitative single cell measurements are given for each of the 11 phosphorylated proteins in each perturbation condition. We normalized the fluorescence signals of the single-cell flow cytometry data against the cell size and against overlapping wavelength ranges of the emission signals of the fluorophores used for the 11 molecules, as described in file S1. We then sampled from the data using bootstrapping to get 10 bootstrap samples with three replicates each from the data. The replicates where then further summarized by taking the median. We inferred a network for each bootstrap sample using the DEPN and LP approaches. Inference with the LP model was carried out with parameters 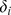 set to the median of the nine measured conditions for each molecule 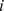. LOOCV was used to determine 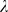, where parameters of the two normal distributions for active and inactive states were determined from measurements of the activated respectively the inactivated molecules. These distributions were then used in the crossvalidation runs to predict left-out protein states required for the MSE computation, and optimal 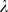 were then used for final network predictions. Edge weights from the LOOCV were summarized across the bootstrap samples using the median. Additional details are given in file S1. Inference using the DEPN approach was performed using greedy hillclimbing and bootstrapping with 100 bootstrap samples for each of the sampled data sets. The median of the inferred edge weights over the samples was used for final evaluation.

### ErbB Signaling in Breast Cancer Cells

As a second evaluation on real data, we used recently published data on ErbB signaling in a breast cancer cell line. The ErbB signaling pathways are some of the best studied signaling networks and it is known that they regulate diverse physiological responses such as cell division, motility and survival [40]. Fröhlich *et al.* focused on the 16 proteins ERBB1, ERBB2, ERBB3, IGF1R, ER-alpha, pAKT1, pERK1/2, MYC, Cyclin D1, p27, p21, Cyclin E1, CDK6, CDK4, CDK2 and pRB1. The proteins are all involved in the ErbB receptor-regulated G1/S cell cycle transition network. For a detailed description of the experimental setup see [28]. In short, the authors used RNAi knockdowns followed by reverse phase protein arrays (RPPA) [41], [42] to quantify protein levels. They performed single-knockdowns of the thirteen proteins ERBB1, IGF1R, ER-alpha, pAKT1, pERK1/2, MYC, Cyclin D1, p27, p21, Cyclin E1, CDK6, CDK4, CDK2 and three double-knockdowns of ERBB1+ERBB2, ERBB2+ERBB3 and ERBB1+ERBB3 with chemically synthesized siRNAs as well as one experiment with mock transfected cells as a negative control. RPPA measurements were done before and twelve hours after EGF stimulation for ten intermediates of the network, namely ERBB1, ERBB2, pAKT1, pERK1/2, Cyclin D1, p27, p21, CDK4, CDK2, pRB1, to quantify their protein expression after each individual perturbation. This was repeated in four technical and three biological replicates, which were normalized by the authors using quantile normalization. The remaining proteins could not be quantified due to lack of antibodies suitable for RPPA.

We preprocessed this data further by summarizing replicate measurements using the arithmetic mean. We then solved the LP model based on the data measured 12 hours after the EGF stimulation, using 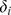 set to the average of the mock control at time zero for the respective protein. We used the constraints (6) with source nodes ERBB1, ERBB2 and ERRB3, and (7) with sink node pRB1. LOOCV was used to estimate 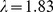.

## Results

We implemented the linear programs in the statistical programming environment R version 2.12.1 [35]. The R cran package “lpSolve” version 5.6.5 was used to solve the linear programs. This package implements the simplex LP solver. The R package “network” was used for graph handling. All calculations were performed on a 3 GHz Intel dual-processor Xeon quadcore computer with 32 GB RAM, running the Linux operating system. No parallelization was used in the computations. Data were simulated as described in methods and analyzed using the network inference approach developed. We studied the effect of different levels of noise and missing values on inference performance on simulated data, as well as effects of overall network size. Results of our linear programming approach were compared with DEPNs as well as random guessing, showing superior performance of our approach. We then applied our method to reconstruct signaling downstream of CD3, CD28 and LFA-1 in CD4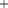 T-cells, as well as signal transduction in the ErbB pathway in breast cancer cells.

### Analysis on Simulated Data

Simulated data allows a systematic evaluation of network inference performance under well defined conditions. The “gold standard” network used to simulate data is known, hence network inference results can directly be evaluated. Furthermore, full control over properties of the data can be exerted, and it is thus possible to systematically study the influence of different levels of noise, missing values, or network size on inference performance. We performed network reconstruction under differing conditions on simulated data, and evaluated results using receiver operator characteristic (ROC) curves and precision-recall (PR) analysis. As a single measurement of inference performance, the area under the ROC and PR curves was used. For all analyses on simulated data, we assumed no further prior knowledge about the underlying network, in particular, we did not specify which of the nodes were source or sink nodes in the network. Constraints (6) and (7) were hence not used.

### Ten-Node Networks

**Figure 1:**
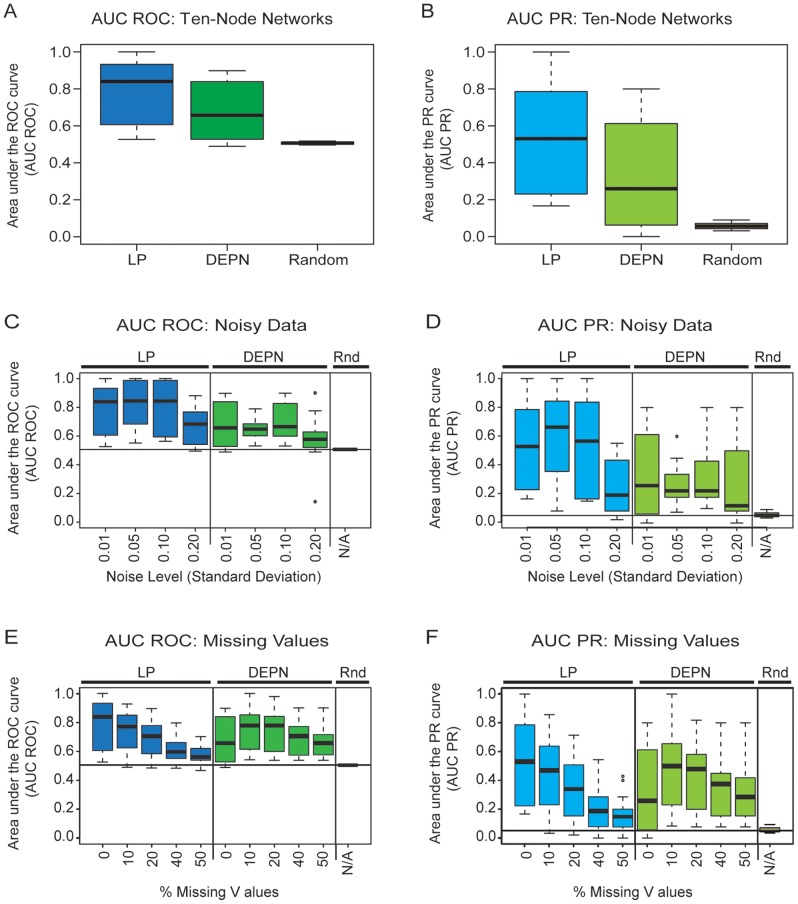
Evaluation on simulated data for small-scale network reconstruction. The figure shows the area under the receiver operator characteristic (AUC ROC) and area under the precision-recall (AUC PR) curves on simulated networks of size ten nodes. Shown are crossvalidation results over 10 simulated data sets, with reconstruction performed using the Linear Program (LP), Deterministic Effects Propagation Networks (DEPN) and random guessing. (A) and (B) show performance on data with low noise (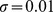) and with no missing values, (C) and (D) illustrate performance effects of increasing levels of noise, and (E) and (F) regard effects of missing values on inference results.

As a first evaluation of our approach, we reconstructed networks from the simulated ten-node data sets, without noise or missing values. Reconstruction was done on averages of three simulated replicates for each of the ten networks, and the area under the ROC and PR curves was calculated for each of the 10 reconstructions. Figures 1A and 1B show the distribution of the AUC ROC and AUC PR values so obtained, respectively, over the 10 simulated networks. We furthermore used the DEPN approach on the same data, and provide its performance for reference in the figures. Furthermore, the rightmost boxplot in the figures shows the achieved performance for random guessing, derived by 100 fold random permutation of edges in the true network – thus guessing a network with the same number of edges as the true network.

We observed superior performance of the LP approach both in terms of the AUC ROC and AUC PR evaluation. This result is somewhat surprising, since the method used for data simulation is closely related to the model assumptions made by the DEPN approach, with a deterministic signal transduction and noise introduced only at the level of the experimental measurements. Variability of performance is comparable across the two methods, with interquartile ranges of approximately 0.3 for the AUC ROC and almost 0.6 for the AUC PR. Both approaches perform significantly better than random guessing, indicating that both methods are able to extract information about the underlying signal transduction networks from the knockdown data. We note that the small values for the AUC PR are due to the fact that the selected sub-networks are all sparse, a property they inherit from the networks stored in the KEGG database.

We next assessed performance of our approach under increasing levels of noise in the experimental data. This was achieved by increasing the variability of the normal distributions used to simulate measurements, as described in methods, using standard deviations 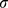 of 0.01, 0.05, 0.1 and 0.2, and again summarizing data from three replicates and assessing performance over ten different networks. Results are shown in figures 1C and D, showing the distribution of achieved AUC ROC and AUC PR values for the LP model, DEPN, and random guessing (Rnd). Guessing is done independently of the actual data, and hence uninfluenced by the level of noise in the data. As expected, performance of both network inference approaches deteriorates with increasing levels of noise, but all approaches remain superior to guessing even for the highest level of noise simulated. We consistently observed better performance of the LP approach, indicating that our method can adequately handle noisy data.

As a further performance test, we evaluated the effect of missing data on reconstruction performance. We left out up to 50% of the data, and reconstructed networks using only the remaining values (figures 1E, F). This resulted in a performance decrease for the LP and the DEPN approach, however, both methods are still better than random guessing even when 50% of the data are missing.

Furthermore, we tested the impact of prior knowledge on network reconstruction performance. For this purpose, we either disclosed the identity of source and sink nodes in the true underlying network by using constraints (6) and (7), or we added additional constraints to force 10%, 25%, 50% or 100% of the true edges in the gold standard network to have weight 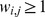, thus requiring the edge to be present in the reconstructed network. As expected, the more prior knowledge we included in the model the better are the resulting predictions, compare figure S2.

To evaluate the performance of our approach on networks having inhibitory interactions, we randomly selected half of the edges of each of the ten-node networks to be deactivating. We simulated data for these networks similarly to the networks having only positive interactions with 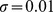. We then applied our inference approach on the data and assessed the performance by computing AUC ROC and AUC PR values (figure S3). In spite of the additional complexity of the three class problem (activation, inhibition or no edge), overall performance is only marginally affected.

### Larger Problems and Runtime Analysis

**Figure 2:**
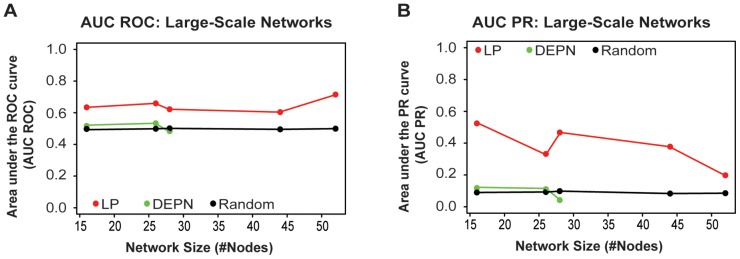
Impact of network size. Effect of network size on network reconstruction – AUC values of the ROC (A) and the PR (B) curves of the network inference using the LP model, the DEPNs and random guessing, for different network sizes. Results were obtained using stratified 10-fold crossvalidation. Calculation with DEPN did not finish within 1000 hours of computation time for networks of size 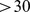, and computations were thus interrupted.

We next assessed performance of our network inference approach on larger networks with 16 to 52 nodes. Data were simulated as described in methods, and network inference was carried out using 10-fold crossvalidation. To summarize results, crossvalidation runs were aggregated by using the median of the crossvalidation runs for each individual edge weight. We compared results with the DEPN approach and with random guessing. Figure 2 shows a comparison of the resulting AUC values from the receiver operator characteristic (2A) and precision-recall analysis (2B). On these larger networks, AUC-ROC values between 0.6 and 0.7 and AUC-PR values around 0.4 were achieved consistently for all network sizes tested using the linear programming approach, whereas the DEPNs were only marginally better than random guessing.

**Figure 3:**
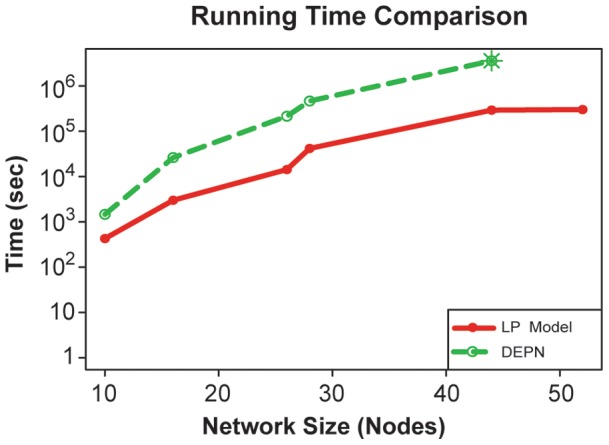
Running time. The figure shows the computation time required to infer networks of different sizes, for the LP (red solid circles and line) and DEPN (green dashed circles and line) approaches, in seconds. Note the logarithmic scale of the y-axis. Computation time is for full evaluation of stratified 10-fold crossvalidation. Computations for networks for 44 and 52 nodes with DEPN were aborted after 1000 hours (green star) without a solution.

To assess runtime performance of the network inference, we measured the average required time to infer the underlying network for our LP model and the DEPN approach. Figure 3 shows the measured running times for the two approaches on networks of increasing size; note the logarithmic scale of the Y-axis. The LP model requires on average 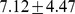 (mean 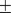 standard deviation) minutes for the ten-node data sets with 16 simulated knockdowns. This is a significant speedup over the DEPN approach on the same network, which requires 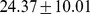 minutes and even yields inferior reconstruction results (Figure 2). For networks of size 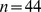 and 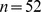, crossvalidation computations with the DEPN approach took over 1000 hours, and were then interrupted. The crossvalidation runs for the corresponding linear program could still be finished within 85 hours, giving an at least 12 fold decreased runtime.

### Evaluation on Flow Cytometry Data

An evaluation on simulated data has the advantage that data properties and simulation conditions can be tightly controlled and a gold standard network for performance evaluation is available. However, only an assessment on real data can ultimately proof practical applicability of an approach in a biological setting. We therefore evaluated network inference performance of our approach on published flow cytometry data, studying 11 phosphorylated proteins and phospholipids downstream of CD3, CD28 and LFA-1 activation in human primary nave CD4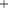 T cells. We compared results obtained using our approach with the network as published by Sachs *et al.* as a reference network [15]. Notably, the Bayesian network approach by Sachs *et al.* exploits individual cell measurements from flow cytometry data, which were summarized to average values for the inference with DEPN and LP model. The amount of data exploited for the inference is thus substantially smaller for the latter two approaches. We then calculated true positive edges (TP), false positive edges (FP), true negative edges (TN) and false negative edges (FN) and used these values to compute sensitivity, specificity, precision and accuracy of the network reconstruction. Table 1 summarizes the results for the LP model, DEPN, the Bayesian approach pursued by Sachs et al., and random guessing. P-values were obtained based on the empirical distribution for guessing. The Bayesian network approach pursued by Sachs et al. achieved superior results for all of the above measures, with an achieved specificity of 95% (p
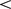
0.00001), sensitivity of 71% (p
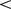
0.00001), precision of 71% (p
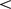
0.00001) and accuracy of 92% (p
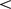
0.00001). This is likely due to the substantially larger amount of data available from using individual cell measurements, which had to be summarized for the DEPN and LP models. Performance of both the LP model and the DEPN approach was inferior to the Bayesian network, with specificity 91% (p
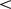
0.00001), sensitivity 18% (p = 0.47), precision 25% (p = 0.1) and accuracy 81% (p = 0.02) for the LP model, and specificity 93% (p
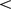
0.00001), sensitivity 12% (p = 0.72), precision 22% (p = 0.32) and accuracy 82% (p = 0.01) for the DEPN approach.

**Table 1:** Evaluation results on T-Cell signaling.

	LP model	LP model REP	DEPN	Sachs *et al.*	random
TP	3	9	2	12	2.72
TN	95	102	97	99	91.72
FP	9	2	7	5	13.28
FN	14	8	15	5	13.28
SP	0.91^**^	0.98^**^	0.93^**^	0.95^**^	0.86
SN	0.18	0.53^**^	0.12	0.71^**^	0.16
PR	0.25	0.82^**^	0.22	0.71^**^	0.16
AC	0.81^*^	0.92^**^	0.82^*^	0.92^**^	0.76

The table shows performance measures for the network inference on flow cytometry data regarding signaling downstream of CD3, CD28 and LFA-1 in CD4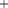 T-cells. Network inference was performed using the linear program (LP), Deterministic Effects Propagation Networks (DEPN), random guessing, and a Bayesian network model as implemented by Sachs *et* *al.* TP  =  true positives, TN  =  true negatives, FP  =  false positives, SP  =  specificity, SN  =  sensitivity, PR  =  precision, AC  =  accuracy. The column “LP model REP” corresponds to the evaluation results of the LP model where the reversely inferred edges and reported indirect regulations are counted as true positives. Statistically significant differences are marked with 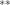
(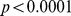) and 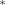
(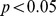), respectively.

Hence, with comparable accuracy, the LP approach achieved higher sensitivity and precision than the DEPN, at the expense of inferior specificity. Notably, if we score only correctly predicted interactions between two proteins, not taking directionality of the interaction into account and consider direct edges that arise from indirect regulations (through intermediate proteins) as correctly scored, the LP model achieves a specificity of 98% (p
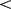
0.00001), sensitivity of 53% (p
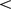
0.00001), precision of 82% (p
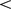
0.00001) and accuracy of 92% (p
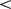
0.00001), achieving higher specificity and higher precision also than the Bayesian network. Table 1 furthermore reports results for random guessing, which were obtained using 100-fold random permutation of edges in the reference network.

**Figure 4:**
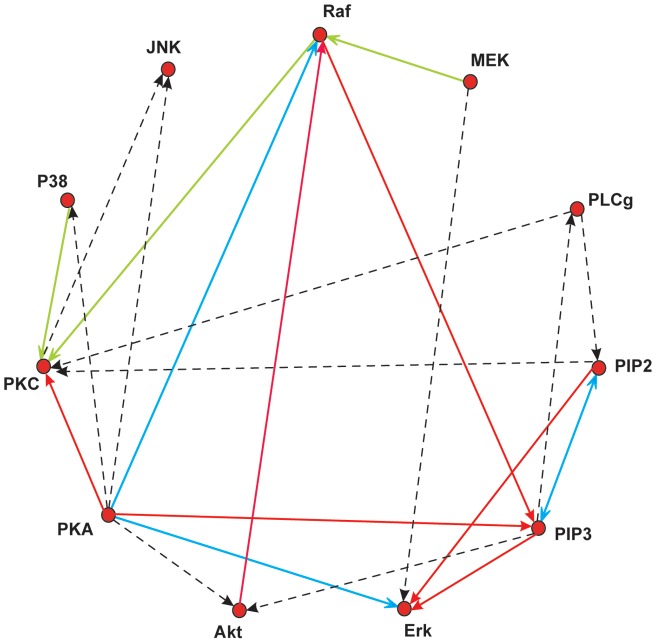
Signaling in CD4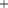 T-cells. LP network inference results of the flow cytometry data. The blue lines correspond to true positive edges given in the reference network from Sachs *et al.* Green edges have been predicted in the wrong direction (reversed edges) and red edges are false positives. Dashed lines are missed interactions.

Reconstruction with our LP approach on the full data set led to the reconstructed network shown in figure 4 (table S2 reports the edge weights). Blue and red edges are true and false positives, respectively, dashed lines correspond to false negative edges and green lines are edges where a link between the two proteins exists, but the directionality of the interaction was predicted incorrectly. Regarding the inferred edges more closely, we observed that the edges from PIP2 to Erk and from PIP3 to Erk inferred with the LP model are given as indirect connections via PKC
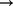
Mek and via PIP2 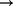
PKC
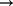
Mek in the reference network. Furthermore, the learned activation of PKC by PKA has already been predicted (albeit in reversed direction) by Sachs *et al.*

### ErbB Signaling is controlled by Negative and Positive Feedback Loops

**Figure 5:**
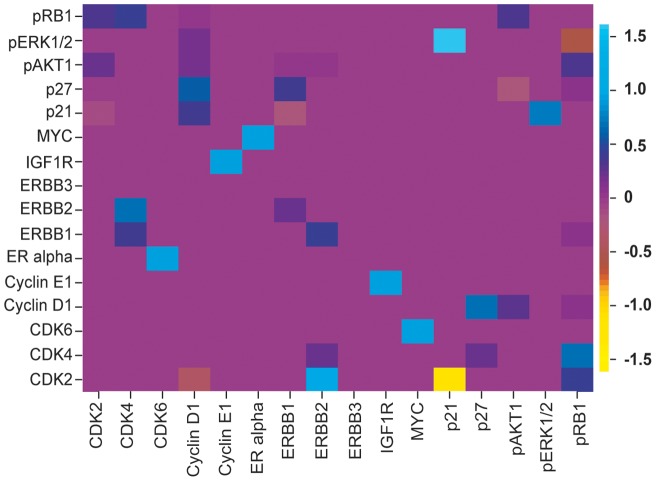
Reconstructed edge weights in ErbB signaling. Imageplot of median of inferred edge weights 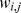 of the ErbB signaling data. Shown are average results from the crossvalidation runs. Parameter 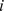 refers to columns and 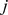 to rows, hence there is for example a strong inhibition of CDK2 by p21.

We next applied our approach to publicly available reverse phase protein array measurements obtained after knockdowns and pathway activation in the ErbB pathway [28]. For a description of data and inference procedure, see methods. The inferred network using the LP approach consists of 43 interactions (34 activations and 9 inhibitions). Figure 5 shows a heatmap of the reconstructed edge weights, where the 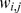 are color coded: pink corresponds to no interaction (zero edge weight), blue to an activation, and yellow to an inhibition. Detailed median edge weights with error bars (median absolute deviation, MAD) are given in table S3. For further analysis, we removed all interactions with coefficient of variation larger than one, thus removing edges with high uncertainty. This procedure resulted in a network with a total of 35 interactions (31 activations, 4 inhibitions), which we analyzed further.

Concerning inhibitory interactions, our inference predicts a strong inhibition of CDK2 by p21, with edge weight 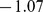. This is a known inhibition that has previously been reported [43]. Furthermore, we inferred deactivations of pERK1/2 by pRB1 and CDK2 by Cyclin D1, with edge weights 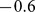 and 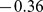, respectively. Both inhibitions seem biologically plausible feedback loops to control the G1/S cell cycle transition, but have not previously been reported. However, there is some evidence showing co-precipitation of CDK2 and Cyclin D1 [44]. Last but not least, we predict an inactivation of p21 by ERBB1 with strength 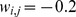. This inhibition can be found in the literature as an indirect path via pAKT1 and MYC [28]. Comparing these results with the DEPN approach run on the same data, we firstly have to emphasize that DEPNs cannot infer negative edge weights, and therefore are not able to directly learn any inhibitory influences. Nevertheless, all of the interactions mentioned above with the exception of the pRB1 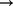 pERK1/2 inhibition have been inferred using the DEPN approach as unsigned interactions.

Regarding activations, the strongest inferred activation of our LP approach is the activation of pERK1/2 by p21, with 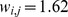. This result suggests the presence of a strong positive feedback loop controlling the G1/S cell cycle transition. Interestingly, albeit no direct activation of ERKs by p21 has been reported previously, it is known that p21 strongly increases the phosphorylation of cFos and MBP by ERK1 and ERK2 [45], thus constituting a feedback on regulatory effects mediated by ERK. We furthermore predict an activation of CDK2 by ERBB2 with weight 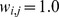. Although the direct connection of these proteins has not been reported in the literature, there exist two indirect signaling paths: ERBB2 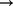 pAkt1 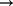 MYC 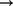 Cyclin E1 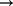 CDK2 and ERBB2 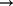 pERK1/2 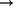 MYC 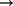 Cyclin E1 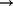 CDK2, which support our results [28]. Our approach furthermore predicts five interactions with edge weights of 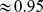 each: MYC activates CDK6, IGF1R activates Cyclin E1 and vice versa, CDK6 activates ERalpha and ERalpha activates MYC. The last activation is known from literature [46]. The connection between IGF1R and Cyclin E1 is known by an indirect path via pERK1/2 and MYC [28]. The two other interactions between MYC and CDK6, and between CDK6 and ERalpha are newly predicted activations. Using the DEPNs on the same data, an indirect path was learned from MYC to CDK6: MYC 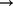 p27 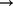 CDK4 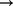 Cyclin D1 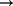 CDK6 [28]. Among the remaining learned activations with lower edge weights, the activations of ERBB1 by ERBB2 and vice versa are worth mentioning, since the two kinases are known to form heterodimers [40], [47].

To evaluate these results further, we used the String database (http://string-db.org) as reference network [48]. We use all interactions of the 16 proteins with a combined confidence score higher than 0.92. Since the interactions given in String are undirected and unsigned, we removed the edge weights of our inferred network topology as well as the signs. We then computed sensitivity, specificity, precision and accuracy for the inferred networks. In addition, we compared results against 1000 randomly generated networks, which were derived by randomly permuting the edges in the String reference network. Last but not least, we compared our results with results of the DEPN approach on the same data, as previously published [28]. We note that an analysis on the transitive closure of the reference network is not possible, since string does not contain any directionality information. Table 2 shows the complete results obtained. Both LP and DEPN achieved a specificity of 83% (p
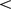
0.00001), while the LP model achieved higher sensitivity (21%, p
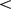
0.00001) than DEPN (14%, p
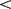
0.00001). In terms of accuracy and precision, LP outperformed DEPN (accuracy: 63%, p = 0.13 LP vs. 60%, p = 0.22 DEPN; precision: 38%, p = 0.44 LP vs. 28%, p = 0.1 DEPN).

**Table 2:** Evaluation results on ErbB signaling.

	LP model	DEPN	random
TP	9	6	14.72
TN	71	71	58.72
FP	15	15	27.28
FN	33	36	27.28
SP	0.83^**^	0.83^**^	0.68
SN	0.21^**^	0.14^**^	0.35
PR	0.38	0.28	0.35
AC	0.63	0.60	0.57

Shown are comparative performance measurements for network inference on reverse phase protein array data regarding ErbB signaling in breast cancer cells. Network inference was performed using our linear programming (LP) approach, Deterministic Effects Propagation Networks (DEPN), and random guessing of a network. Results were compared with a gold standard network from the String database. TP  =  true positives, TN  =  true negatives, FP  =  false positives, SP  =  specificity, SN  =  sensitivity, PR  =  precision, AC  =  accuracy. Statistically significant differences are marked with 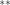
(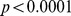) and 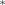
(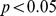), respectively.

In conclusion, we learned several already known activations and inactivations and inferred potential new interactions. The most interesting new predictions are probably those which indicate negative or positive feedback loops, since they allow it to regulate and control the G1/S cell cycle transition. Chen *et al.* showed that the ERBB response is silenced by negative feedback from active ERK [47], supporting the idea of feedback loops in ERBB signaling.

## Discussion and Conclusions

With the availability of large-scale experimental datasets and easy and relatively inexpensive access to perturbation experiments, functional screens offer a direct means to elucidate cellular signaling in living cells. However, the reconstruction of signal transduction networks from perturbation data is a challenging problem that, in spite of increased attention in the last decade, still is in desperate need for novel algorithms. The problem has been shown to be NP complete even if a core topology is known, and only minimal changes to make a model consistent with experimental data are sought for [32]. Various statistical and machine learning approaches have been developed to reconstruct networks from observational data, and several address network inference from perturbation experiments. Main challenges in the field come from the complexity of the inference problem, with an exponentially growing number of possible network topologies for increasing network size. Methods such as Nested Effects Models or Deterministic Effects Propagation Networks then quickly reach computational limits when larger networks are targeted.

Our main contribution in this manuscript is the formulation of the network inference problem in terms of an information flow through a graph. While the well known max-flow/min-cut problem in graph theory searches for a maximum flow through a given network, our problem here is inverse in the sense that we know values of the flow through the network for different cuts (knockdowns), and wish to reconstruct the underlying network topology from this data. Using maximum parsimony as a guiding principle, we show how this leads to a formulation of the problem as a linear program, a class of optimization problems that has received considerable attention in combinatorial optimization. Integer linear programming is NP hard, but by making the assumption that the edge weights 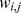 are continuous, we can formulate the network inference problem as a non-integral LP, thus making the problem solvable in polynomial time. Using a heuristic, threshold-based discretization of edge weights, we then arrive at an approximately optimal network topology. This trick allows it to solve substantially larger network inference problems, and present limitations in network inference then no longer arise from the computational complexity, but rather limited availability of perturbation data with sufficient observations of gene/protein activity levels after all knockdowns.

Data requirements for such network inference are still limiting the application of such approaches as ours on large-scale screens. For a screen with 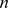 genes, we ideally would need single knockdowns of all 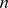 genes, each with subsequent measurements of the activity levels of all 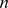 affected proteins. While large-scale screens are widely available, the second requirement – observations of activity levels of all proteins after each knockdown – is still rare and not routinely measured. Microarrays have been used to measure transcriptional activity after gene knockdown, but offer only a very indirect view about changes at the protein level [49]. To complicate matters further, cellular networks are often robustly designed [50], and single knockdowns may not be sufficient to affect cellular phenotypes in such situations due to redundancies in the cellular pathways. This can be overcome by combinatorial screens with double or multiple knockdowns, which can easily be integrated into our LP formulation in a canonical way, but further increase data requirements [51].

Albeit the approach we pursue here is using a deterministic model of signal transduction and pathway activity, we show that it can deal extremely well with noisy, stochastic data. The introduction of slack variables in the formulation of the optimization problem here is key to cope with experimental and biological variability, and permits solution of the model even in the presence of conflicting data. The principle of maximum parsimony, i.e. minimization of the overall sum of edge weights, further helps to drive solutions to sparse networks, and makes the inference feasible even in the presence of substantial amounts of missing data. The formulation of the objective function as we use it, with minimization of the sum of absolute edge weights instead of the sum of squared weights, leads to solutions of the LP where 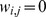 for as many tuples 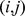 as possible, and hence sparse networks. This is known as Lasso regularization, and is a direct analogue of using a zero-mean Laplace prior in Bayesian network inference, as can immediately be seen by taking the negative logarithm of the Laplace prior. Correspondingly, the parameter 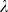 in equation (3) has an intimate relation to the dispersion parameter of the Laplace distribution, and hence relates the expected variance of the weight parameters 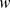 to the variance of the slack variables 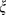. This parameter effectively trades off variability in the experimental data and thus the slack variables to variability in the model parameters 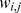. This implies that the value of 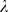 should be chosen not only based on network size, but also taking into account the overall variance of the experimental measurements.

A critical issue in setting up the linear program is the choice of threshold parameters 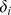 for discretization of gene activity levels. This parameter determines, for each gene 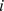 separately, from which level of activation 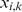 a gene or protein 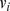 is considered active or inactive. Based on this discretization, either constraint (4) or (5) are used. Correspondingly, the choice of 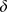 may critically affect results of the whole inference procedure. Optimally, 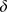 should be determined experimentally from control experiments directly knocking down each gene 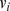 and comparing its activity with a negative control. Alternatively, statistical approaches using significance levels for false positive calls could be used to define a threshold after z-score normalization of the data, for example using the full screen as a quasi negative control [52]. A simple approach that we used in this manuscript was to calculate 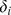 from the data, for example as the mean of the 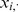 for all experimental measurements of a given gene 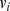.

The simulation study we performed illustrates stable performance of our approach both in light of noisy and missing data, with superior performance over the DEPN approach at least on the noisy data we simulated. On the two real experimental data sets, the LP approach shows higher sensitivity and precision, at the expense of decreased specificity in comparison to the DEPN approach. Since network inference predictions should be considered as hypotheses only and not ultimate reality, and clearly need further experimental validation to firmly establish interactions between involved proteins, it may be an advantage to rather have a higher rate of false positive predictions than too many false negatives.

Importantly, there are fundamentally different assumptions made in the underlying network models of the DEPN and LP approaches. To illustrate the difference, assume a simple network with three nodes 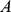, 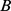, 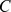, and edges 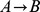, 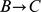, and 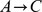 to be given. Furthermore, assume that a knockdown of node 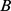 is made. DEPNs now assume that the effect of the knockdown of node 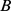 can be observed at node 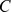, since 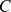 is downstream of 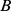. In terms of “activation” of a signaling molecule, this means that DEPNs assume that node 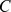 will only become activated, if all of its parents are activated, hence incoming edges are connected with *AND* in the activation function. In the given example, node 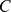 is not activated, due to the knockdown of 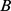. In our model, in the same situation, we assume that node 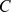 *will* be activated after knockdown of 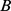, due to the direct edge 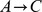. Incoming edges to a node are thus aggregated using the *OR* function. Interestingly, these differences in assumptions make it possible for the LP approach to infer whether or not an edge 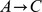 exists, whereas it is impossible to make any statement about this edge using the DEPN model, since under this model, there is no difference in the observed state of node 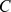, no matter whether the edge 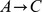 exists or not. It is possible to change these underlying assumptions and implement DEPNs with an “OR” activation function; however, the LP model becomes a nonlinear model if “AND” interactions are assumed.

Our results not only show how the choice of method to be used depends on the research question at hand, e.g. hypothesis generation versus prediction of high confidence interactions, but also on the type and structure of the underlying data: DEPNs are suitable for small-scale networks with large amounts of available data, but rapidly deteriorate with increasing levels of noise. Furthermore, the two models assume different underlying mechanisms of signal transduction and effect propagation. The scenario where the LP approach can play its strengths are larger network inference problems with up to several dozens of genes, possibly involving missing values and higher levels of noise in the data, and ideally comprising single and combinatorial knockdowns. Both approaches require direct readouts of states of the proteins involved in the signaling network, for example from protein arrays. If only indirect observations at the transcriptomic level e.g. through microarrays or RNA sequencing technology are available, or if cellular phenotypes are observed e.g. from microscopy based screening approaches, Nested Effects Models are the method of choice in case of high-dimensional downstream readouts [21]–[25], or Probabilistic Boolean Threshold Networks in case of low-dimensional observations [17]. Neither DEPN in their original form nor our LP approach can directly handle time series data, which would help to reconstruct in particular feedback cycles in signaling networks. D-DEPNs are an extension of DEPNs that are specifically designed for time series experiments [29]. D-DEPNS work particularly well if long time series measurements are available for small networks. Importantly, for small time courses of large networks as tested in this manuscript, D-DEPNs are not applicable and could not be used. An obvious extension of the current LP approach therefore is to explicitly take time series measurements into account and exploit the information conveyed by the temporal evolution of a network to refine network reconstruction.

An interesting observation is the very good performance of the Bayesian network used by Sachs *et al.* on the flow cytometry study [15]. Importantly, the authors use pairwise correlations of the single-cell data to predict dependencies and causal interactions. This additional information coming from hundreds of individual cell measurements cannot be exploited by the DEPN and LP approaches, for which the relative amounts of correlated measurements need to be transformed into an observation matrix which reflects the effects of each perturbation at the average level per knockdown. Thereby, the single-cell information are lost. Integrating such single cell data into combinatorial optimization approaches to network inference is an open issue for future work.

## Supporting Information

Figure S1 **Evaluation on simulated data against transitively closed reference network.** The figures show the area under the receiver operator characteristic (AUC ROC) and area under the precision-recall (AUC PR) curves on simulated ten-node and large-scale networks. Shown are the results for the Deterministic Effects Propagation Networks (DEPN) and random guessing of the transitively closed reference networks. (A) and (B) show performance on data with increasing levels of noise, and (C) and (D) illustrate performance effects of increasing levels of missing data for the ten-node networks. (E) and (F) show the AUC values for the large-scale networks.
(TIF)

Figure S2 **Prior knowledge.** The figure shows the AUC values of (**A**) ROC and (**B**) PR curves of the network inference using the LP model and random guessing on data simulated for the ten-node networks randomly selected from KEGG. The x-axis labeling denotes the percentage of interactions which are defined to be known a priori in the LP model. The LP-SF model is the model with known source and sink nodes.
(TIF)

Figure S3 **Evaluation with inhibitory edges.** The figure shows the evaluation results of a two class ROC analysis considering only activating edges and a three class ROC analysis considering activating and inhibitory edges, as described [20]. The two class results correspond to the **A** ROC and **B** PR curves of the network inference using the LP model and random guessing on data simulated for the ten-node networks randomly selected from KEGG. For the three class evaluation we randomly set half of the edges given in each of the ten-node networks to be inhibitory. We inferred the underlying networks and computed the AUC values as described. The results are shown for the (**A**) ROC and (**B**) PR curves for the three class evaluation on the two boxplots of the right side of each figure. Note that in the three class analysis, random guessing has an AUC ROC value different from 0.5, and a PR value smaller than in the two class case. The dashed horizontal lines show the expected values for random guessing.
(TIF)

Table S1
**Evaluation of the DEPNs with transitively closed reference network.** The table shows performance measures for the network inference on the flow cytometry data regarding signaling downstream of CD3, CD28 and LFA-1 in CD4^+^ T-cells. Network inference was performed using the Deterministic Effects Propagation Networks (DEPN) and random guessing of the transitively closed reference network (reported in Sachs *et al.*). TP  =  true positives, TN  =  true negatives, FP  =  false positives, SP  =  specificity, SN  =  sensitivity, PR  =  precision, AC  =  accuracy. Statistically significant differences are marked with ^**^(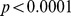) and ^*^(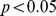), respectively.(PDF)

Table S2 **Inferred edge weights flow cytometry data.** Table S2 shows the average edge weights across the inferred network topologies using the LP model and the bootstrapping approach on the flow cytometry data.
(PDF)

Table S3 **Inferred edge weights ERBB data.** Table S3 shows the median edge weights ± the median absolute deviation (MAD) of all LOOCV-steps for the inferred network topologies, using the LP model on the ErbB signaling data. If the MAD is not given explicitly, it is equal to zero.
(PDF)

Supplementary File S1 **Methods and implementation details.** This pdf-file provides additional information on how the ten-node and large-scale networks have been extracted from KEGG, and it gives details on the preprocessing of the flow cytometry data.
(PDF)

## References

[1] Boutros M, Kiger AA, Armknecht S, Kerr K, Hild M, et al (2004) Genome-wide rnai analysis of growth and viability in drosophila cells. Science 303: 832–835. 14764878 10.1126/science.1091266

[2] Kittler R, Putz G, Pelletier L, Poser I, Heninger AK, et al (2004) An endoribonuclease-prepared sirna screen in human cells identifies genes essential for cell division. Nature 432: 1036–1040. 15616564 10.1038/nature03159

[3] Agaisse H, Burrack LS, Philips JA, Rubin EJ, Perrimon N, et al (2005) Genome-wide RNAi screen for host factors required for intracellular bacterial infection. Science 309: 1248–1251. 16020693 10.1126/science.1116008

[4] Furlong EE (2005) A functional genomics approach to identify new regulators of wnt signaling. Dev Cell 8: 624–626. 15866154 10.1016/j.devcel.2005.04.006

[5] Muller P, Kuttenkeuler D, Gesellchen V, Zeidler MP, Boutros M (2005) Identification of jak/stat signalling components by genome-wide rna interference. Nature 436: 871–875. 16094372 10.1038/nature03869

[6] Friedman A, Perrimon N (2006) A functional rnai screen for regulators of receptor tyrosine kinase and erk signalling. Nature 444: 230–234. 17086199 10.1038/nature05280

[7] Whitehurst AW, Bodemann BO, Cardenas J, Ferguson D, Girard L, et al (2007) Synthetic lethal screen identification of chemosensitizer loci in cancer cells. Nature 446: 815–819. 17429401 10.1038/nature05697

[8] Brass AL, Dykxhoorn DM, Benita Y, Yan N, Engelman A, et al (2008) Identification of host proteins required for HIV infection through a functional genomic screen. Science 319: 921–926. 18187620 10.1126/science.1152725

[9] Krishnan MN, Ng A, Sukumaran B, Gilfoy FD, Uchil PD, et al (2008) Rna interference screen for human genes associated with west nile virus infection. Nature 455: 242–245. 18690214 10.1038/nature07207 PMC3136529

[10] Chia NY, Chan YS, Feng B, Lu X, Orlov YL, et al (2010) A genome-wide rnai screen reveals determinants of human embryonic stem cell identity. Nature 468: 316–320. 20953172 10.1038/nature09531

[11] Collinet C, Stöter M, Bradshaw CR, Samusik N, Rink JC, et al (2010) Systems survey of endocytosis by multiparametric image analysis. Nature 464: 243–249. 20190736 10.1038/nature08779

[12] Börner K, Hermle J, Sommer C, Brown NP, Knapp B, et al (2010) From experimental setup to bioinformatics: an rnai screening platform to identify host factors involved in hiv-1 replication. Biotechnol J 5: 39–49. 20013946 10.1002/biot.200900226

[13] Theis M, Buchholz F (2011) High-throughput rnai screening in mammalian cells with esirnas. Methods 53: 424–429. 21185384 10.1016/j.ymeth.2010.12.021

[14] Moffat J, Sabatini DM (2006) Building mammalian signalling pathways with RNAi screens. Nat Rev Mol Cell Biol 7: 177–187. 16496020 10.1038/nrm1860

[15] Sachs K, Itani S, Carlisle J, Nolan GP, Pe'er D, et al (2009) Learning signaling network structures with sparsely distributed data. J Comput Biol 16: 201–212. 19193145 10.1089/cmb.2008.07TT PMC3198894

[16] Hill SM, Lu Y, Molina J, Heiser LM, Spellman PT, et al (2012) Bayesian inference of signaling network topology in a cancer cell line. Bioinformatics 28: 2804–2810. 22923301 10.1093/bioinformatics/bts514 PMC3476330

[17] Kaderali L, Dazert E, Zeuge U, Frese M, Bartenschlager R (2009) Reconstructing signaling pathways from RNAi data using probabilistic Boolean threshold networks. Bioinformatics 25: 2229–2235. 19542154 10.1093/bioinformatics/btp375

[18] Böck M, Ogishima S, Tanaka H, Kramer S, Kaderali L (2012) Hub-centered gene network reconstruction using automatic relevance determination. PLoS One 7: e35077. 22570688 10.1371/journal.pone.0035077 PMC3343044

[19] Rice JJ, Tu Y, Stolovitzky G (2005) Reconstructing biological networks using conditional correlation analysis. Bioinformatics 21: 765–73. 15486043 10.1093/bioinformatics/bti064

[20] Mazur J, Ritter D, Reinelt G, Kaderali L (2009) Reconstructing nonlinear dynamic models of gene regulation using stochastic sampling. BMC Bioinformatics 10: 448. 20038296 10.1186/1471-2105-10-448 PMC2811124

[21] Markowetz F, Bloch J, Spang R (2005) Non-transcriptional pathway features reconstructed from secondary effects of RNA interference. Bioinformatics 21: 4026–4032. 16159925 10.1093/bioinformatics/bti662

[22] Markowetz F, Kostka D, Troyanskaya OG, Spang R (2007) Nested effects models for highdimensional phenotyping screens. Bioinformatics 23: i305–312. 17646311 10.1093/bioinformatics/btm178

[23] Fröhlich H, Fellmann M, Sültmann H, Poustka A, Beissbarth T (2008) Estimating large-scale signaling networks through nested effect models with intervention effects from microarray data. Bioinformatics 24: 2650–2656. 18227117 10.1093/bioinformatics/btm634 PMC2579711

[24] Tresch A, Markowetz F (2008) Structure learning in Nested Effects Models. Stat Appl Genet Mol Biol 7: Article9. 10.2202/1544-6115.1332 18312214

[25] Fröhlich H, Tresch A, Beissbarth T (2009) Nested effects models for learning signaling networks from perturbation data. Biom J 51: 304–323. 19358219 10.1002/bimj.200800185

[26] Anchang B, Sadeh MJ, Jacob J, Tresch A, Vlad MO, et al (2009) Modeling the temporal interplay of molecular signaling and gene expression by using dynamic nested effects models. Proc Natl Acad Sci USA 106: 6447–6452. 19329492 10.1073/pnas.0809822106 PMC2672479

[27] Frohlich H, Praveen P, Tresch A (2011) Fast and efficient dynamic nested effects models. Bioinformatics 27: 238–244. 21068003 10.1093/bioinformatics/btq631

[28] Frohlich H, Sahin O, Arlt D, Bender C, Beissbarth T (2009) Deterministic effects propagation networks for reconstructing protein signaling networks from multiple interventions. BMC Bioinformatics 10: 322. 19814779 10.1186/1471-2105-10-322 PMC2770070

[29] Bender C, Henjes F, Fröhlich H, Wiemann S, Korf U, et al (2010) Dynamic deterministic effects propagation networks: learning signalling pathways from longitudinal protein array data. Bioinformatics 26: i596–i602. 20823327 10.1093/bioinformatics/btq385 PMC2935402

[30] Ourfali O, Shlomi T, Ideker T, Ruppin E, Sharan R (2007) SPINE: a framework for signalingregulatory pathway inference from cause-effect experiments. Bioinformatics 23: i359–366. 17646318 10.1093/bioinformatics/btm170

[31] Lan A, Smoly IY, Rapaport G, Lindquist S, Fraenkel E, et al (2011) Responsenet: revealing signaling and regulatory networks linking genetic and transcriptomic screening data. Nucleic Acids Res 39: W424–W429. 21576238 10.1093/nar/gkr359 PMC3125767

[32] Hashemikhabir S, Ayaz ES, Kavurucu Y, Can T, Kahveci T (2012) Large scale signaling network reconstruction. IEEE/ACM Trans Comput Biol Bioinform. 10.1109/TCBB.2012.128 23221085

[33] Khachiyan LG (1979) A polynomial algorithm in linear programming. Dokl Akad Nauk SSSR 244: 1093–1096.

[34] Schrijver A (1999) Theory of Linear and Integer Programming. Wiley.

[35] R Core Team (2012) R: A Language and Environment for Statistical Computing. R Foundation for Statistical Computing, Vienna, Austria.

[36] Leclerc RD (2008) Survival of the sparsest: robust gene networks are parsimonious. Mol Syst Biol 4: 213. 18682703 10.1038/msb.2008.52 PMC2538912

[37] Kanehisa M, Goto S (2000) KEGG: kyoto encyclopedia of genes and genomes. Nucleic Acids Res 28: 27–30. 10592173 10.1093/nar/28.1.27 PMC102409

[38] Fawcett T (2006) An introduction to roc analysis. Pattern Recognition Letters 27: 861–874.

[39] Sachs L, Hedderich J (2006) Angewandte Statistik: Methodensammlung mit R. Springer.

[40] Citri A, Yarden Y (2006) EGF-ERBB signalling: towards the systems level. Nat Rev Mol Cell Biol 7: 505–516. 16829981 10.1038/nrm1962

[41] Tibes R, Qiu Y, Lu Y, Hennessy B, Andreeff M, et al (2006) Reverse phase protein array: validation of a novel proteomic technology and utility for analysis of primary leukemia specimens and hematopoietic stem cells. Mol Cancer Ther 5: 2512–2521. 17041095 10.1158/1535-7163.MCT-06-0334

[42] Charboneau L, Tory H, Scott H, Chen T, Winters M, et al (2002) Utility of reverse phase protein arrays: applications to signalling pathways and human body arrays. Brief Funct Genomic Proteomic 1: 305–315. 15239896 10.1093/bfgp/1.3.305

[43] Harper JW, Elledge SJ, Keyomarsi K, Dynlacht B, Tsai LH, et al (1995) Inhibition of cyclindependent kinases by p21. Mol Biol Cell 6: 387–400. 7626805 10.1091/mbc.6.4.387 PMC301199

[44] Bates S, Bonetta L, MacAllan D, Parry D, Holder A, et al (1994) CDK6 (PLSTIRE) and CDK4 (PSK-J3) are a distinct subset of the cyclin-dependent kinases that associate with cyclin D1. Oncogene 9: 71–79. 8302605

[45] Neise D, Sohn D, Budach W, Jänicke RU (2010) Evidence for a differential modulation of p53-phosphorylating kinases by the cyclin-dependent kinase inhibitor p21waf1/cip1. Cell Cycle 9: 3575–3583. 20818156 10.4161/cc.9.17.12799

[46] Sutherland RL, Prall OW, Watts CK, Musgrove EA (1998) Estrogen and progestin regulation of cell cycle progression. J Mammary Gland Biol Neoplasia 3: 63–72. 10819505 10.1023/a:1018774302092

[47] Chen WW, Schoeberl B, Jasper PJ, Niepel M, Nielsen UB, et al (2009) Input-output behavior of ErbB signaling pathways as revealed by a mass action model trained against dynamic data. Mol Syst Biol 5: 239. 19156131 10.1038/msb.2008.74 PMC2644173

[48] Szklarczyk D, Franceschini A, Kuhn M, Simonovic M, Roth A, et al (2011) The string database in 2011: functional interaction networks of proteins, globally integrated and scored. Nucleic Acids Res 39: D561–D568. 21045058 10.1093/nar/gkq973 PMC3013807

[49] Boutros M, Agaisse H, Perrimon N (2002) Sequential activation of signaling pathways during innate immune responses in Drosophila. Dev Cell 3: 711–722. 12431377 10.1016/s1534-5807(02)00325-8

[50] Stelling J, Sauer U, Szallasi Z, Doyle FJ 3rd, Doyle J (2004) Robustness of cellular functions. Cell 118: 675–85. 15369668 10.1016/j.cell.2004.09.008

[51] Horn T, Sandmann T, Fischer B, Axelsson E, Huber W, et al (2011) Mapping of signaling networks through synthetic genetic interaction analysis by rnai. Nat Methods 8: 341–6. 21378980 10.1038/nmeth.1581

[52] Rieber N, Knapp B, Eils R, Kaderali L (2009) RNAither, an automated pipeline for the statistical analysis of high-throughput RNAi screens. Bioinformatics 25: 678–679. 19168909 10.1093/bioinformatics/btp014

