# Supplementary figures and images for: Reconstruction of Cellular Signal Transduction Networks Using Perturbation Assays and Linear Programming
Source: PLoS One. 2013 Jul 30;8(7):e69220. doi: 10.1371/journal.pone.0069220 (PMC3728289; doi:10.1371/journal.pone.0069220)

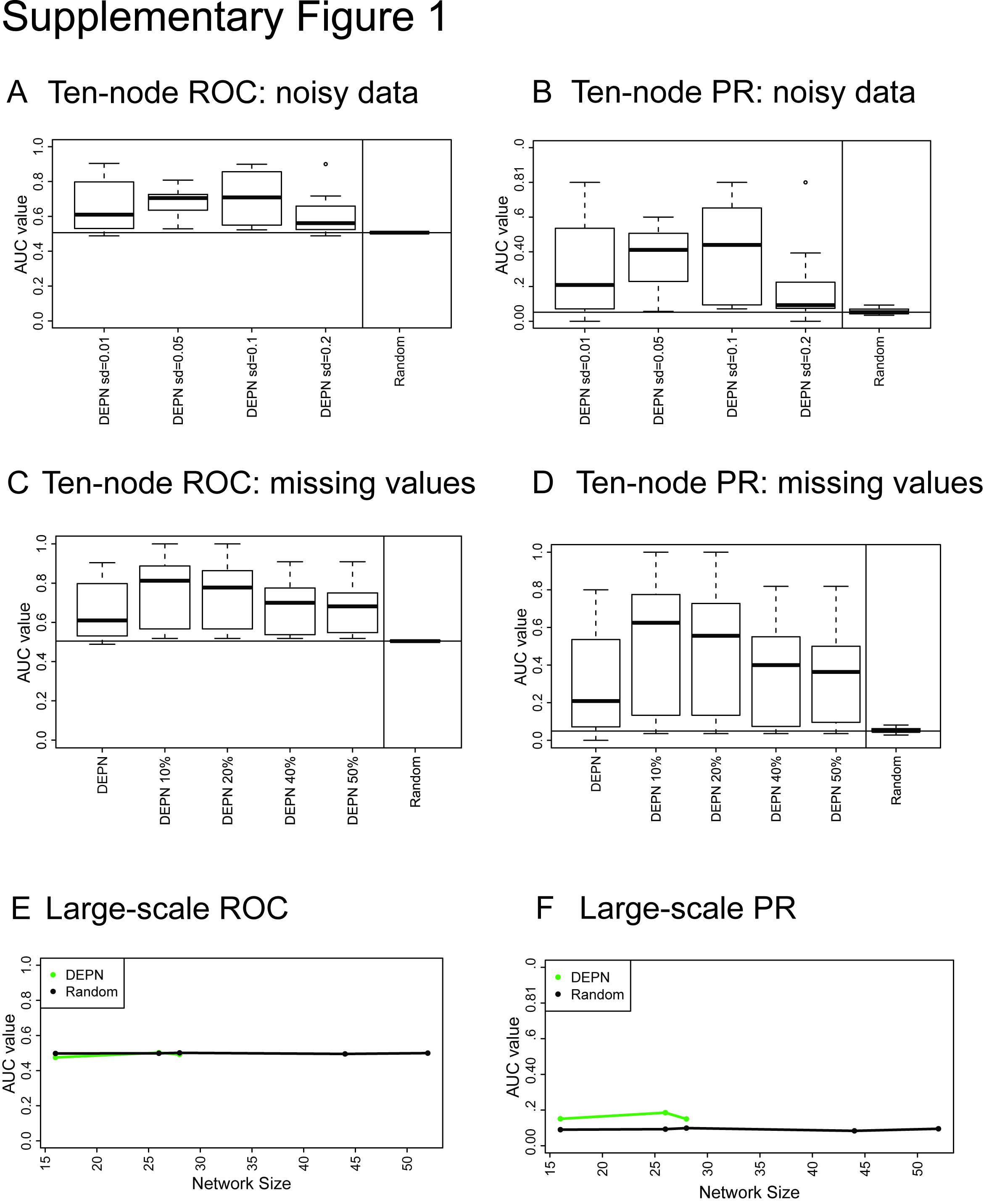

Supplement: Figure S1 — Evaluation on simulated data against transitively closed reference network. The figures show the area under the receiver operator characteristic (AUC ROC) and area under the precision-recall (AUC PR) curves on simulated ten-node and large-scale networks. Shown are the results for the Deterministic Effects Propagation Networks (DEPN) and random guessing of the transitively closed reference networks. (A) and (B) show performance on data with increasing levels of noise, and (C) and (D) illustrate performance effects of increasing levels of missing data for the ten-node networks. (E) and (F) show the AUC values for the large-scale networks. (TIF) [file pone.0069220.s001.tif]

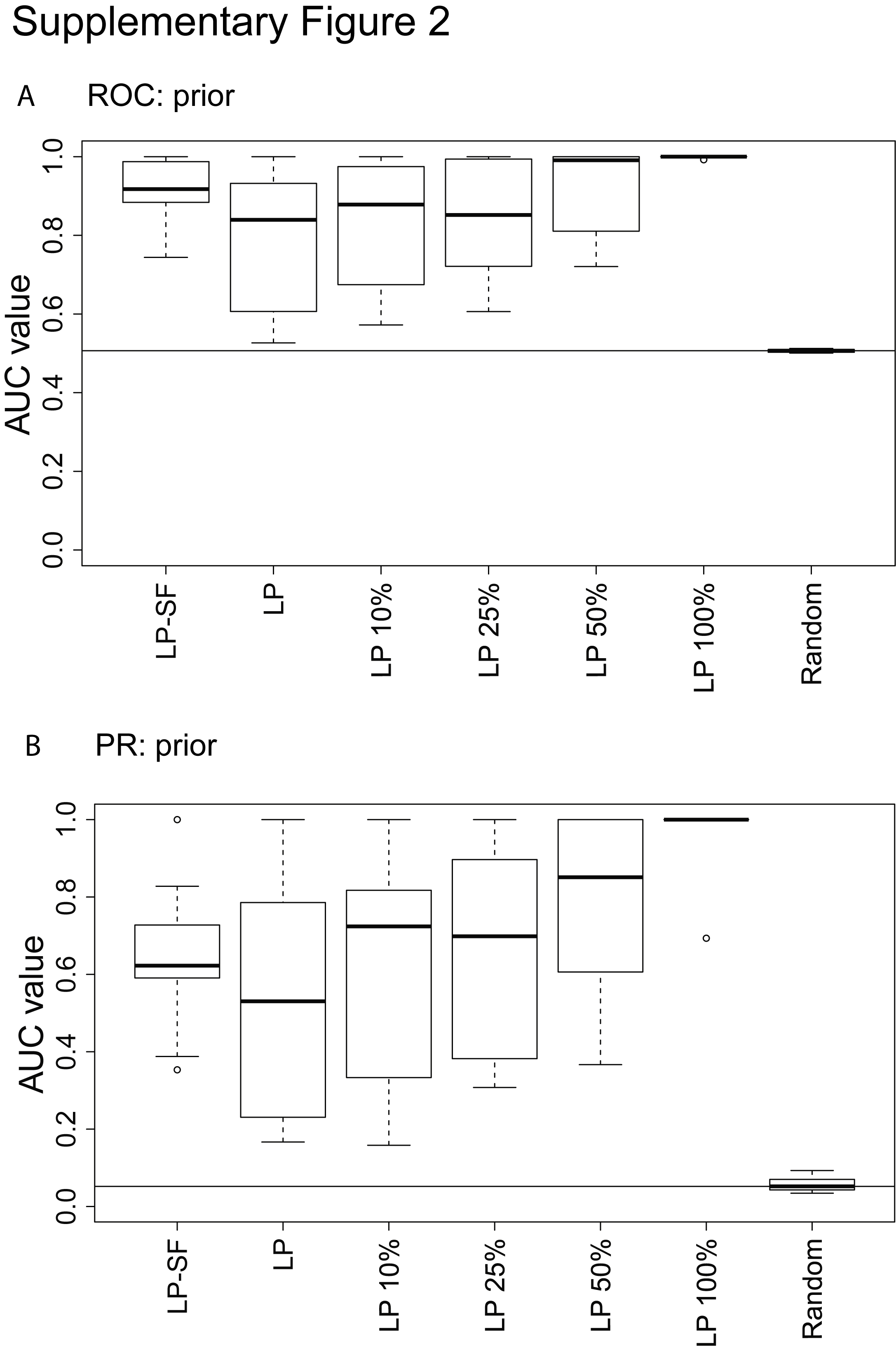

Supplement: Figure S2 — Prior knowledge. The figure shows the AUC values of (A) ROC and (B) PR curves of the network inference using the LP model and random guessing on data simulated for the ten-node networks randomly selected from KEGG. The x-axis labeling denotes the percentage of interactions which are defined to be known a priori in the LP model. The LP-SF model is the model with known source and sink nodes. (TIF) [file pone.0069220.s002.tif]

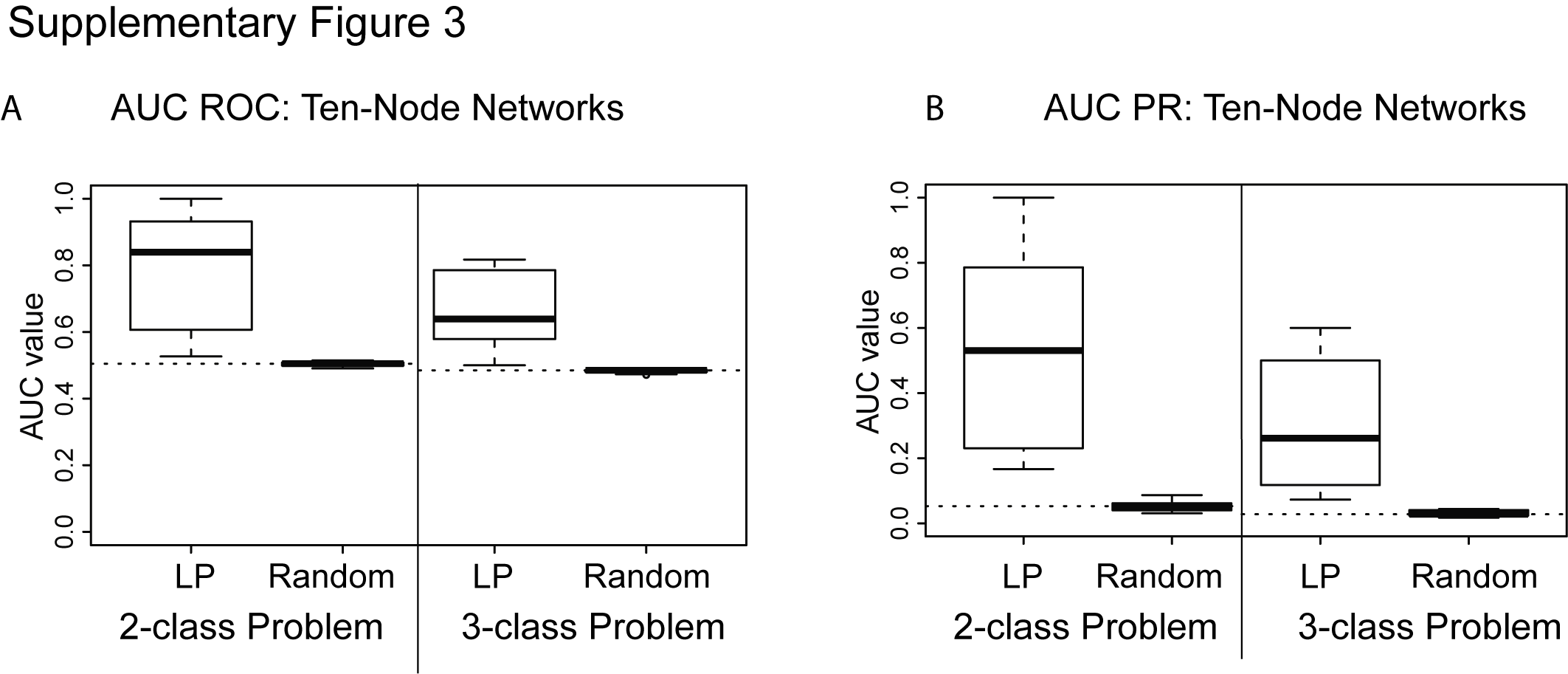

Supplement: Figure S3 — Evaluation with inhibitory edges. The figure shows the evaluation results of a two class ROC analysis considering only activating edges and a three class ROC analysis considering activating and inhibitory edges, as described [20]. The two class results correspond to the A ROC and B PR curves of the network inference using the LP model and random guessing on data simulated for the ten-node networks randomly selected from KEGG. For the three class evaluation we randomly set half of the edges given in each of the ten-node networks to be inhibitory. We inferred the underlying networks and computed the AUC values as described. The results are shown for the (A) ROC and (B) PR curves for the three class evaluation on the two boxplots of the right side of each figure. Note that in the three class analysis, random guessing has an AUC ROC value different from 0.5, and a PR value smaller than in the two class case. The dashed horizontal lines show the expected values for random guessing. (TIF) [file pone.0069220.s003.tif]
