# Supplementary material for: Reconstruction of Cellular Signal Transduction Networks Using Perturbation Assays and Linear Programming
Source: PLoS One. 2013 Jul 30;8(7):e69220. doi: 10.1371/journal.pone.0069220 (PMC3728289; doi:10.1371/journal.pone.0069220)
Supplement: Table S1 — Evaluation of the DEPNs with transitively closed reference network. The table shows performance measures for the network inference on the flow cytometry data regarding signaling downstream of CD3, CD28 and LFA-1 in CD4+ T-cells. Network inference was performed using the Deterministic Effects Propagation Networks (DEPN) and random guessing of the transitively closed reference network (reported in Sachs et al.). TP = true positives, TN = true negatives, FP = false positives, SP = specificity, SN = sensitivity, PR = precision, AC = accuracy. Statistically significant differences are marked with **() and *(), respectively. (PDF) [file pone.0069220.s004.pdf]

Supplementary Table 1: Evaluation results on T-Cell signaling using the transitively closed true network. TP = true positives, TN = true negatives, FP = false positives, SP = specificity, SN = sensitivity, PR = precision, AC = accuracy. The values which are significantly different to random with a p-value < 0.0001, respectively < 0.05 are marked with **, respectively with *.

	**DEPN**	**random**
TP	5	15.32
TN	76	54.32
FP	4	25.68
FN	36	25.68
SP	0.95^{**}	0.68
SN	0.12^{**}	0.37
PR	0.56^{**}	0.37
AC	0.67^{*}	0.58
