# Supplementary material for: Reconstruction of Cellular Signal Transduction Networks Using Perturbation Assays and Linear Programming
Source: PLoS One. 2013 Jul 30;8(7):e69220. doi: 10.1371/journal.pone.0069220 (PMC3728289; doi:10.1371/journal.pone.0069220)
Supplement: Table S2 — Inferred edge weights flow cytometry data. Table S2 shows the average edge weights across the inferred network topologies using the LP model and the bootstrapping approach on the flow cytometry data. (PDF) [file pone.0069220.s005.pdf]

Supplementary Table 2: Average of the edge weights w_{ij} inferred in the bootstrap steps of the flow cytometry data using the LP model. The edge weight w_{ij} corresponds to an edge between genes of the ith row and the jth column.

	Raf	Mek1/2	PLYγ	PIP2	PIP3	Erk1/2	Akt	PKA	PKC	p38	JNK
Raf	0	0	0	0	0.06	0	0	0	0.02	0	0
Mek1/2	0.04	0	0	0	0	0	0	0	0	0	0
PLYγ	0	0	0	0	0	0	0	0	0	0	0
PIP2	0	0	0	0	0.07	0.02	0	0	0	0	0
PIP3	0	0	0	0	0	0.02	0	0	0	0	0
Erk1/2	0	0	0	0	0	0	0	0	0	0	0
Akt	0.02	0	0	0	0	0	0	0	0	0	0
PKA	0.17	0	0	0	0.13	0.25	0	0	0.09	0	0
PKC	0	0	0	0	0	0	0	0	0	0	0
p38	0	0	0	0	0	0	0	0	0.01	0	0
JNK	0	0	0	0	0	0	0	0	0	0	0
