# Supplementary material for: Reconstruction of Cellular Signal Transduction Networks Using Perturbation Assays and Linear Programming
Source: PLoS One. 2013 Jul 30;8(7):e69220. doi: 10.1371/journal.pone.0069220 (PMC3728289; doi:10.1371/journal.pone.0069220)
Supplement: Table S3 — Inferred edge weights ERBB data. Table S3 shows the median edge weights ± the median absolute deviation (MAD) of all LOOCV-steps for the inferred network topologies, using the LP model on the ErbB signaling data. If the MAD is not given explicitly, it is equal to zero. (PDF) [file pone.0069220.s006.pdf]

Supplementary Table 3: Median±MAD of inferred edge weights w_{ij} using the LP model on the ErbB signaling data where i corresponds to genes of the ith row and j to genes of the jth column. If the MAD is not given explicitly, it is equal to zero.

	CDK2	CDK4	CDK6	Cyclin D1	Cyclin E1	ERalpha	ERBB1	ERBB2	ERBB3	IGF1R	MYC	p21	p27	pAKT1	pERK1/2	pRB1
CDK2	0	0	0	0	0	0	0	0	0	0	0	-0.14±0.21	0	0.25±0.37	0	0.33±0.27
CDK4	0	0	0	0	0	0	0.4±0.05	0.69	0	0	0	0	0	0	0	0.44±0.18
CDK6	0	0	0	0	0	0.95	0	0	0	0	0	0	0	0	0	0
Cyclin D1	-0.36±0.05	0	0	0	0	0	0	0	0	0	0	0.39±0.02	0.59±0.32	0.16±0.06	0.18±0.03	0.01±0.02
Cyclin E1	0	0	0	0	0	0	0	0	0	0.95	0	0	0	0	0	0
ERalpha	0	0	0	0	0	0	0	0	0	0	0.95	0	0	0	0	0
ERBB1	0	0	0	0	0	0	0	0.21	0	0	0	-0.2±0.07	0.36	0.03±0.05	0	-0.04±0.06
ERBB2	1±0.07	0.21±0.05	0	0	0	0	0.42±0.06	0	0	0	0	0	0	0	0	-0.02±0.03
ERBB3	0	0	0	0	0	0	0	0	0	0	0	0	0	0	0	0
IGF1R	0	0	0	0	0.95	0	0	0	0	0	0	0	0	0	0	0
MYC	0	0	0.95	0	0	0	0	0	0	0	0	0	0	0	0	0
p21	-1.07±0.15	0	0	0	0	0	0	0	0	0	0	0	0	0	1.62±0.03	0
p27	0	0.25	0	0.7	0	0	0	0	0	0	0	0	0	0	0	0
pAKT1	0	0	0	0.26	0	0	0	0	0	0	0	0	-0.2±0.3	0	0	0.31±0.15
pERK1.2	0±0.01	0	0	0	0	0	-0.02±0.02	0	0	0	0	0.72±0.14	0	0	0	0
pRB1	0.45±0.11	0.67±0.05	0	0.05	0	0	0.06	0	0	0	0	0	0.06±0.08	0.35±0.19	-0.6±0.06	0
