# Supplementary material for: Reconstruction of Cellular Signal Transduction Networks Using Perturbation Assays and Linear Programming
Source: PLoS One. 2013 Jul 30;8(7):e69220. doi: 10.1371/journal.pone.0069220 (PMC3728289; doi:10.1371/journal.pone.0069220)
Supplement: Supplementary File S1 — Methods and implementation details. This pdf-file provides additional information on how the ten-node and large-scale networks have been extracted from KEGG, and it gives details on the preprocessing of the flow cytometry data. (PDF) [file pone.0069220.s007.pdf]

Supplementary Material File 1

Reconstruction of Cellular Signal Transduction Networks using Perturbation Assays and Linear Programming

Bettina Knapp[1,2] and Lars Kaderali[1,2]

[1]Technische Universität Dresden, Medical Faculty Carl Gustav Carus, Institute for Medical Informatics and Biometry, Fetscherstrasse 74, 01307 Dresden, Germany
[2]Heidelberg University, ViroQuant Research Group Modeling, BioQuant BQ26, Im Neuenheimer Feld 267, 69120 Heidelberg, Germany
`bettina.knapp@tu-dresden.de`

1 Networks extracted from KEGG

For the evaluation of the LP model, we randomly extracted sub-networks from different KEGG pathways (KEGG IDs: hsa05212, hsa05210, hsa04630, hsa04370, hsa04350, hsa04310, hsa04210, hsa04115, hsa04110, hsa04012, hsa04010) [1]. We randomly selected one of these KEGG IDs. Then, we randomly selected genes from the network of this ID using the package KEGGgraph [3] from bioconductor `http://www.bioconductor.org`. We repeated this process until we extracted ten networks with ten nodes each. The selected sub-networks do not have to be fully connected, but each of the selected genes has to have at least one interaction to one of the other randomly selected genes. We used these ten-node networks to evaluate the performance of our approach on noisy and missing data.
Furthermore, we randomly extracted networks of larger sizes. For this, we selected 60 genes from the pathways of five randomly selected KEGG IDs. Then, we removed the genes which do not have an interaction to one of the other selected nodes. Thereby, we extracted five sub-networks with 16,26,28,44 and 52 nodes and used them to quantify how the computational time increases with increasing network size.

2 Flow Cytometry Data

2.1 Perturbation conditions

Data published by Sachs *et al.* [2] was collected for nine different perturbation conditions including four activations:

1. Anti-CD3/CD28: activates T cells and induces proliferation and cytokine production.
2. ICAM-2: initiates LFA- signaling and contributes to CD3/CD28 signaling.
3. PMA: activates PKC and induces some aspects of T cell signaling.
4. β2cAMP: activates PKA.

Furthermore, there are five inhibitions:

1. AKT inhibitor: inhibits AKT.
2. U0126: inhibition of MEK1/2.
3. G06976: inhibits PKC and arrests T cell activation.
4. Psitectorigenin: inhibition of PIP2.
5. LY294002: PI3K inhibitor and prevents activation of AKT.

See Sachs *et al.* [2] for more details.

2.2 Data normalization

We normalized the data by subtracting from each fluorescence intensity the median of the fluorescence intensities of the 11 molecules (PKC, PKA, Akt, Raf, Mek1/2, Erk1/2, p38, JNK, PIP2, PIP3, PLYγ) divided by the MAD of these 11 molecules for each cell. This normalization accounts for differences in the signal intensities of different cells due to different cell sizes. Then, we summarized the fluorescence signals of each molecule $i = 1, \ldots, 11$ by taking the median across all cells for each of the $j = 1, \ldots, 9$ perturbation experiments. This results in an observation matrix with measurements for each of the 11 phosphorylated proteins and phospholipids after each perturbation.
To compensate for overlapping wavelength ranges of the emission signals of the fluorophores used for the 11 molecules we performed further normalizations. For this, we first shift the data to positive values by subtracting the smallest signal intensity from all others. Then, we sort the mean signal intensities $\bar{s}_i = (\sum_j s_{ij})/9$ for each molecule i (measured with a specific fluorophore) in decreasing order. Starting with the largest $\bar{s}_i$, we normalize the intensities s_{ij} of the respective fluorophore i if $\bar{s}_i > 1$. If $\bar{s}_i > \bar{s}_{/i}$ (with $\bar{s}_{/i}$ the mean of all observations except those of i) we compute the normalized intensity s_{ij}^{norm} with $s_{ij}^{norm} = (\bar{s_i}/\bar{s_{/i}}) * \bar{s_i}$. Otherwise, we set $s_{ij}^{norm} = (\bar{s_{/i}}/\bar{s_i}) * \bar{s_i}$.
Using a bootstrapping approach on the normalized data, we sampled three “replicates” of the data in 10 bootstrap samples. For this, we randomly select one third of the cells of each condition. The median signals of these cells of each of the 11 molecules correspond to one replicate measurement.

2.3 Computation of the Gaussian parameters

The parameters of the two Gaussian distributions coming from activated, respectively inactivated genes are determined by the genes which are active, respectively inactive. The Gaussian of the activated state is given by the median

and MAD of the observed measurements of Raf, PIP2 and PIP3 (which are activated by CD3 and CD28) after the 1. and 2. activation experiment, the observed measurement of PKC after the 3. activation experiment, and the observed measurement of PKA after the 4. activation experiment. The Gaussian of the inactivated state is given by the median and MAD of all $b_{ik} = 0$ with $i = 1, \ldots, 11$ and $k = 1, \ldots, 9$ (thus, all observations of silenced molecules).

References

1. M. Kanehisa and S. Goto. KEGG: kyoto encyclopedia of genes and genomes. *Nucleic Acids Res.*, 28:27–30, Jan 2000.
2. K. Sachs, S. Itani, J. Carlisle, G. P. Nolan, D. Pe'er, and D. A. Lauffenburger. Learning signaling network structures with sparsely distributed data. *J. Comput. Biol.*, 16(2):201–212, Feb 2009.
3. Jitao David Zhang and Stefan Wiemann. *KEGGgraph: KEGGgraph: A graph approach to KEGG PATHWAY in R and Bioconductor*, 2009. R package version 1.2.2.
